# Guidance in Social and Ethical Issues Related to Clinical, Diagnostic Care and Novel Therapies for Hereditary Neuromuscular Rare Diseases: “Translating“ the Translational

**DOI:** 10.1371/currents.md.f90b49429fa814bd26c5b22b13d773ec

**Published:** 2013-01-10

**Authors:** Pauline McCormack, Simon Woods, Annemieke Aartsma-Rus, Lynn Hagger, Agnes Herczegfalvi, Emma Heslop, Joseph Irwin, Janbernd Kirschner, Patrick Moeschen, Francesco Muntoni, Marie-Christine Ouillade, Jes Rahbek, Christoph Rehmann-Sutter, Francoise Rouault, Thomas Sejersen, Elizabeth Vroom, Volker Straub, Kate Bushby, Alessandra Ferlini

**Affiliations:** Newcastle University, Newcastle, United Kingdom; Newcastle University, Newcastle, United Kingdom; Leiden University Medical Center, Leiden, The Netherlands; University of Sheffield, Sheffield, United Kingdom; Semmelweis Medical University, Budapest, Hungary; Newcastle University, Newcastle, United Kingdom; Lakeside Regulatory Consulting Services Ltd, United Kingdom; University Medical Center Freiburg, Freiburg, Germany; Parent Project Muscular Dystrophy, London, United Kingdom; University College London Institute of Child Health/Great Ormond Street Hospital for Children, London , United Kingdom; Association Française contre les Myopathies, Evry, France; Danish National Rehabilitation Centre for Neuromuscular Diseases, Århus, Denmark & European NeuroMuscular Center, (ENMC), Amsterdam, The Netherlands; University of Lübeck, Lübeck ,Germany; Association Française contre les Myopathies, Evry, France; Karolinska Institutet, Stockholm, Sweden; United Parent Project Muscular Dystrophy (UPPMD), The Netherlands; Newcastle University, Newcastle, United Kingdom; Newcastle University, Newcastle, United Kingdom; University of Ferrara, Ferrara, Italy

## Abstract

Drug trials in children engage with many ethical issues, from drug-related safety concerns to communication with patients and parents, and recruitment and informed consent procedures. This paper addresses the field of neuromuscular disorders where the possibility of genetic, mutation-specific treatments, has added new complexity. Not only must trial design address issues of equity of access, but researchers must also think through the implications of adopting a personalised medicine approach, which requires a precise molecular diagnosis, in addition to other implications of developing orphan drugs. It is against this background of change and complexity that the Project Ethics Council (PEC) was established within the TREAT-NMD EU Network of Excellence. The PEC is a high level advisory group that draws upon the expertise of its interdisciplinary membership which includes clinicians, lawyers, scientists, parents, representatives of patient organisations, social scientists and ethicists. In this paper we describe the establishment and terms of reference of the PEC, give an indication of the range and depth of its work and provide some analysis of the kinds of complex questions encountered. The paper describes how the PEC has responded to substantive ethical issues raised within the TREAT-NMD consortium and how it has provided a wider resource for any concerned parent, patient, or clinician to ask a question of ethical concern. Issues raised range from science related ethical issues, issues related to hereditary neuromuscular diseases and the new therapeutic approaches and questions concerning patients rights in the context of patient registries and bio-banks. We conclude by recommending the PEC as a model for similar research contexts in rare diseases.

## Introduction

In EU countries a rare disease (RD) is any disease affecting fewer than 5 people in 10,000 1 2 which translates to approximately 425,000 people throughout the EU's 27 member countries. (www.eucerd.eu and Orphanet database www.orpha.net). More than 80% of these diseases are caused by genetic defects. Screening for early diagnosis, followed by suitable care, can improve quality of life and life expectancy. Due to the limited interest of pharmaceutical industry in developing and marketing products, the EU and national governments have prioritised the development of “orphan drugs” for patients with rare diseases. Of the 7,000 rare diseases collected in the Online Mendelian Inheritance in Man (OMIM) database (http://www.ncbi.nlm.nih.gov/omim) only 3,000 genes are known, despite the fact that medical genetics has clearly shown that a prerequisite to approaching diagnosis and cure for a RD is to identify the causative mutated gene.

As joint effort will maximise the success and reduce the cost of developing therapies for RDs, it is therefore crucial to co-operate within a national and supranational context and this has been recognised via the formation of a novel, co-operative initiative by the EU, Canada and the USA, the International Rare Diseases Research Consortium (IRDiRC) (IRDiRC, http://ec.europa.eu/research/health/medical-research/rare-diseases/irdirc_en.html).

The objective of the IRDiRC is to deliver diagnostic tests for most RDs and 200 new therapies for RD patients by 2020.

Among RDs, neuromuscular diseases (NMDs) are a major research focus. There are a total of 250,000 patients in Europe with an estimated NMD frequency of 150-200 per 100,000 3 4 . NMDs have gained such attention for a number of reasons including: i) relatively high frequency of NMDs, thought to be around 3% of all RDs; ii) progressive course and devastating impact on the quality of life; iii) elevated mortality often at an early age; iv) very high costs in terms of social and health care.

For these reasons RDs and in particular NMDs are considered pivotal for the EU in the field of translational research as evidenced by a raft of important goals and initiatives also related to RDs registries, biobanks, repositories, site for public consultations and to address the search for excellenct laboratories for the genetic diagnoisis, novel trials and care of the disease (http://www.rdtf.org/testor/cgi-bin/OTmain.php; http://www.orpha.net/; http://www.eurordis.org/; http://asso.orpha.net/RDPlatform/upload/file/SummaryReportRDPlatf03Dec09.pdf).

The challenge of tackling the problem of RDs involves the co-ordination at international level of strategies operating on different fronts including: the creation of a common care pathway; research on diagnostics; establishment of standards of care; collation of patient data through disease registries; and a co-ordinated international network of contact with communication between patients, clinicians, scientists and industry, on the premise that joint effort will maximise success and reduce costs.

NMDs can be caused by mutations in hundreds of different genes and the rarity and severity of NMDs varies, but they invariably lead to serious impairment and loss of autonomy. Although there is a history of clinical trials dating back to the 1970s, it is only during the last decade that the first clinical trials for therapies for genetic disease in humans finally arrived 5 6 7 8 9 10 11 12 13 14.

TREAT-NMD is a Network of Excellence founded by the EU within FP6 (www.treat-nmd.eu). It was designed to provide exactly the joined-up network described above. TREAT-NMD aims to advance diagnosis and care and develop new treatments for the benefit of patients and families, working closely with scientists, healthcare professionals, pharmaceutical companies and patient groups around the world. TREAT-NMD either directly, through its work or indirectly, through supporting various satellite projects on RDs, has succeeded in initiating or supporting initiatives on diagnosis, standards of care, patient registries, operating procedures and models, clinical trial sites, patient communication and outcome measures.

Work on a wide set of issues across such complex terrain will encounter numerous ethical issues and TREAT-NMD established a Project Ethics Council (PEC), a multidisciplinary group comprised of clinicians, scientists, ethicists, legal academics, parents of patients and representatives of parent and patient organisations, in order to respond to such issues. The PEC was established as a high level, multidisciplinary advisory group able to respond to ethical questions arising from within the network, and provide a strategic steer on ethical issues for the Governing Board. It was also recognised that the PEC needed to be outward facing and accessible to the whole of the TREAT-NMD network, from those engaged in translational work to family members and patients. As the PEC established itself as a well-formed group operating to clear and mutually agreed terms of reference it also widened its remit by being accessible to anyone with an interest in NMDs, rather than just to TREAT-NMD members. The TREAT-NMD website therefore became an invaluable resource, providing an access point for individuals or organisations to pose a question and as a place where results of the PEC’s deliberations could be publicly posted (see http://www.treat-nmd.eu/resources/ethics/pec/questions-received/).

This paper aims at reporting the PEC activities within TREAT-NMD and would be a point of reference for others seeking to establish a similar PEC model. In addition to extolling a model of good ethical governance we draw attention to the kinds of question raised in the PEC. We believe these are exemplars of common types of ethical concern across the wider context of RDs. By drawing attention to these concerns we emphasise the necessity, within complex clinical research programmes, to provide the resources and expertise to address ethical issues directly and substantively. An important aspect in the added value of the PEC is its diverse membership of stakeholders from the NMD field, which allows differing perspectives on a problem in a way that speaks authentically to the ethical concerns of NMD patients and families.

## Establishing an Ethics Council

The PEC was constituted as a high-level advisory group and although there is considerable expertise shared across the members of the PEC it was never envisaged that the PEC would be the source of formal or technical guidance on points of law, regulation and governance for the project. The PEC is rather a forum for discussion and debate concerning emerging ethical issues and a source of "reflective wisdom" for those posing particular questions. The underpinning principles of the PEC, in keeping with a number of contemporary bioethics approaches sought to establish a group that was inclusive, democratic and deliberative. The source of the PEC’s reflective wisdom therefore stemmed in part from members who are bioethicists and academic lawyers used to bringing intellectual analysis to ethical problems, but also from the empirical wisdom of clinicians, scientists, parents of children with NMDs and from individuals with careers spanning scientific research and work with patient organisations. The PEC’s deliberations produced written responses to questions and other papers made available on the TREAT-NMD web site.

The PEC quite quickly adopted a deliberative reflexive approach as its method for considering the ethical issues brought before it. Although some may consider this version of an ethics council to be a luxury within the tight budget lines of a funded project we found that the PEC could operate highly effectively and very economically by making use of simple technology such as the TREAT-NMD intranet and a closed email list, with email exchanges and conference calling proving very effective for discussion and deliberation.

## Terms of reference

A key issue for the PEC was to create terms of reference that were neither too restrictive nor too permissive, yet captured the key functions of the Council. Rather than adopting a policing function the PEC placed itself as responsive to issues as they were raised by anyone within and outside the network.

Perhaps the most substantive ethical issue dealt with within the terms of reference concerned the question of confidentiality and here it was agreed that priority ought to be given to openness, meaning that the business of the PEC could be openly discussed outside of the PEC unless there was explicit agreement that an issue of particular sensitivity was required to be treated in confidence. It was also agreed that PEC members should take appropriate care to ensure that any public statements, oral and written presentations on behalf of the PEC would be made in the spirit of the PEC’s terms of reference and clearly distinguished from any statements members made in a personal capacity. Thus, the terms of reference provided some rules of thumb for its remit but allowed for flexibility and the potential for an evolving role within TREAT-NMD. From the very outset it was clear that the PEC was to be used as a sounding-board on a wide range of ethical issues covering all aspects of the TREAT-NMD network. Over time it became clear that substantive discussion within the PEC was driven in two ways, one was by questions posed by non-PEC members and the other was by issues brought to the table by PEC members, often acting as conduits for concerns and questions raised by the wider NMD community with an interest in advancing the cause of neuromuscular disease care and treatment.

On establishment the work of the Council was viewed as somewhat experimental. Professional experience of the members meant terms of reference and procedures for working were soon agreed. However, setting up a dedicated, consultative question and service for empirical ethics within a distributed research network was novel to most TREAT-NMD members, including the PEC and the decision to work in a reflexive manner developed as the PEC established its position. Instituting formal metrics for the PEC’s effectiveness was not therefore seen as compatible with such exploratory methodology. That the PEC’s work was seen as helpful, constructive and effective is shown by the fact that the Council was consulted regularly on ethical matters and was recommended by word of mouth throughout the network. There were also requests from colleagues for the PEC to provide additional resources, for example with a publicly available online guide to stem cell treatment and cord blood banking (http://www.treat-nmd.eu/sma/clinical-research/stem-cell-tourism/).

## Issues

There was a shared common starting point, with which all PEC members agreed, that since NMDs cause a great deal of suffering in children (and their families) there is a moral imperative to advance new therapies. This is a basic ethical tenet of the TREAT-NMD PEC. It did not however lead PEC members to see all medical research as equally necessary, to underestimate the side-effects of treatments, or to support clinical trials uncritically. PEC members also agreed that NMD patients have a right that appropriate clinical trials happen which might lead to an improvement of their situation.

The TREAT-NMD PEC did not operate within a vacuum but rather against a background of need and growing frustration that the fragmentation of translational research on rare disease was resulting in delays that were directly detrimental to patients. Patient organisations, some of which pre-date the establishment TREAT-NMD by several decades, had already done much to organise themselves, to galvanise interest in RD research and to become informed and effective lobbyists 15 . The PEC therefore entered the scene when there was an existing organised and complex community of interest. It is apparent from the questions received by the PEC that a major preoccupation of the community was clinical trials: when, where, how, and for whom?

In the following section we outline some of the central concerns brought to the PEC for its consideration. Table I reports on some of the questions that have been posed to the PEC during its 5 years of activity.

It is hoped that by reporting the issues and questions that came to the PEC together with the PEC’s responses will provide a general picture of the major common concerns related to NMDs and to RDs more generally.

**Table I. Selection of questions posed to the PEC during its 5 years of activity:** 

Issue	Submitted by	Responses
**Relationships with industry:** How can the network protect its own interests at the same time as managing multiple relationships with companies who may be competitors?	Clinician	Fruitful relationships with industry should not be jeopardised by adopting either a too restrictive approach or by a failure to safeguard the genuine interests of industry partners. There is a need to be open and honest with industry, noting the need for transparency without compromising commercially sensitive information.Patient registries should be open to access by industry partners and researchers and the Steering Committee for the registries ought to act as an intermediary to ensure ethical access to and use of data. See http://www.treat-nmd.eu/resources/ethics/pec/questions-received/#1
**Steriods in clinical trials:** Steroids are accepted as standard treatment for DMD but steroids are also recognised as a confounding factor in assessing efficacy of new molecules. Is it ethical to have trials where the boys are not on steroids?	Clinician	Since steroid use has become an international standard therapy it would be wrong to withdraw a beneficial treatment for research purposes. However not all patients have been exposed to steroids and therefore this information ought to be collated on the patient registries so as to be available for investigators of clinical trials. See http://www.treat-nmd.eu/resources/ethics/pec/questions-received/#2
**Carrying patients over from one trial phase to another:** In rare disease studies there can be a limited number of patients available for trial participation. Is it ethical to ask the same patients to take part in more than one phase of a trial?	Clinician	An adequate response to this question would always be to a large extent ‘question specific’, ‘disease specific’, ‘intervention specific’, and (always) ‘protocol specific’. The particular features of the case will tell whether a patient would have an increased risk or any other additional burden. There is a real danger of creating a Catch 22 situation where a population qualifies for inclusion in research by virtue of having a rare disease but is at the same time disqualified from research due to the risk of over research. There may also be a further case on what might be called social or moral grounds that whilst it may not be scientifically necessary to use the same cohort it is fair and respectful (e.g. of autonomy) to do so only where there is little or no risk. See http://www.treat-nmd.eu/resources/ethics/pec/questions-received/#6
**Unproven stem cell “treatment”:** What advice should be given to a patient who is considering paying for stem cell therapy at a centre which claims to be able to treat muscular dystrophy?	Clinician	This is a highly topical and complex issue and one that raises questions about patient autonomy, paternalism, global governance, hope and hype regarding new biotechnologies. There is no question that patients, or the parents of young patients are potential consumers within a globalised market. However, the ethics of the market place can not be the ethics of the clinic and the provider of “goods and services” does not have a fiduciary duty to protect the interests and welfare of the patient in the same way that their clinician does. A key basis of autonomy is knowledge: It is a necessary condition of an autonomous choice that parents/patients are able to judge the current state of knowledge. Using an intervention where the risks are unknown or not made known, and without the evidence for benefit makes it impossible to make an informed choice. This is not informed consent but rather blind faith. See http://www.treat-nmd.eu/resources/ethics/pec/questions-received/#7
**Guaranteeing a place on a clinical trial:** Is it possible for a patient to guarantee a place on a clinical trial by paying to take part?	Parent/relative of patient	The very idea of buying a place on a clinical trial suggests a complete misunderstanding about the purposes of a trial and an unrealistic expectation about its outcome. A trial is the therapeutic testing of a potential treatment for humans; it is not a treatment or a cure. Trials are not treatment; they are designed to test for toxicity and side effects and/or to demonstrate whether the drug will have the desired effect. They allow the collection of scientific data about a group, not about a single individual. In addition, according to the European Clinical Trials Directive 2001/20/EC it is unethical for any clinician or scientist to act in such a way. Such actions are also regarded as illegal in many countries. See http://www.treat-nmd.eu/resources/ethics/pec/questions-received/#8
**Use of patient registries by industry:** How much information should companies be required to make available to patients about the selection of trial sites, particularly when the company has used patient registries to help with selection?	Patient organisation/parent	The PEC endorses the need for open and transparent communication between those accessing patient data via the registries and patient organisations but does not endorse the imposition of restrictive conditions which might prove to be counter-productive for the global registry in its relations with industry and researchers. The PEC does not agree that there is a right for patients to be involved in a clinical trial although it strongly endorses the right for clinical trials to happen in the most efficient and timely manner. In the interests of fairness and equity there should be effort to build the capacity for research to take place in every partner country. See http://www.treat-nmd.eu/resources/ethics/pec/questions-received/#9
**N=1 trials**: Is it ethical to offer personalised therapies to individual patients, before clinical trials of the therapy have taken place?	Scientist	There is currently no proof that long term treatment with the therapy in question - antisense oligonucleotides to induce exon skipping - is eﬀective and safe as placebo-controlled trials have not yet been conducted. The placebo effect can not be discounted and in addition not much is known about longer term tolerability and toxicity of these compounds, especially in children and adolescents. N = 1 trials are not trials in the correct sense of the word. Rather they are the administration of potentially harmful substances to a patient in the (possibly unjustiﬁed) hope that it might help, but without evidence that there are reasonable chances that it will. There would be a serious risk of setting the entire ﬁeld back should anything go wrong. The requests for exon skipping treatment for individual patients arise from therapeutic misconception and everyone in the field has a responsibility to prevent raising false hope and unrealistic expectations. Obviously, by agreeing to conduct trials in single patients, clinicians only increase misconception and also – should exon skipping fail to work as hoped – increase the sense of loss and disappointment. See http://www.duchenne.nl/1214_artaartsmaNMDn=1
**Patient representation:** How should the patient voice be represented within TREAT- NMD management and governance structure?	Patient organisation	There are two aspects to this question – the constitution of TREAT-NMD and its structure. There must be an executive committee that is fit for purpose as well as much wider mechanisms for facilitating the patient voice throughout TREAT-NMD. It is critical that TREAT-NMD develops a structure that is synergistic with the aims and goals of patients, which must include a mechanism to collect, focused views and opinions. On the matter of ensuring that the patient voice is encouraged and facilitated throughout the Alliance then the PEC would encourage the questioner (UPPMD) to provide TREAT-NMD with advice and support regarding the good practices and approaches that have proven successful in the past. See http://www.treat-nmd.eu/resources/ethics/questions-received/#10

## Trial data sharing and timeliness of clinical trials

TREAT-NMD took as its initial focus two of the commonest forms of NMD in childhood, Spinal Muscular Atrophy (SMA) and Duchenne Muscular Dystrophy (DMD). Both are severe, chronic diseases following a relentless progressive course characterised by wheelchair and ventilator reliance, thus giving parents an acute awareness of the time factor in developing new therapies. It has become well established from other RDs that early treatment of progressive disease is more effective 16 .

Several members of the PEC with connections to patient organisations expressed concern that a significant hindrance to clinical trial development was the delayed publication of trial results in peer reviewed journals. Such complaints are not merely expressions of frustration but supported by evidence which has shown the critical connection between the public reporting of trial results and the speed at which new trials are developed. 17 18 19 . A contributing factor to this information bottleneck is the fact that by far the majority of RD trials are sponsored by pharmaceutical companies who, as investors, are naturally cautious about issues of data privacy and intellectual property 20 . These issues, together with the timing required for getting a trial approved by regulatory authorities, may delay the release of data into the public domain via peer reviewed journals. Furthermore, trials can produce large amounts of data, which takes time to collate and analyse. The challenge of analysis can also be complicated by the fact that many participants in RD trials are children affected with chronic diseases, which increases the complexity of interpreting the outcome 21 22 .

Notwithstanding these problems there remain important ethical and practical imperatives in RDs. Clinical trials in RDs always involve a limited (and sometimes very limited) number of patients, which can mean offering novel drugs in personalised, mutation specific, experimental treatments. Due to the chronic, progressive nature of DMD and SMA and other NMDs trials should be long enough to be able to show clinical amelioration measured by clinical outcome measures and clinical endpoints 23 24 . This is why it is highly desirable that trial outcome results are available to other researchers as quickly as possible, in order to inform further work and to minimize wasted efforts 25 .

There is therefore a need to promote rapid publication of data from clinical trials in RDs, but also to facilitate debate around clinical trial results in order to inform new research strategies.

These imperatives will require a culture change both in the attitudes of pharma and the approaches adopted by the academic community. Ideally trial data should be published through the peer review system under which academic journals operate, in preference to the current phenomena of trial results often being solely reported on pharma’s own websites or in press releases, under their own editorial scrutiny. However there are also problems with the very system we recommend, peer reviewed academic journals can take a very long time to review and publish a paper. Journals reporting on clinical trials should adopt a common and comprehensive system of fast-track review and publishing of trial data. Publishers and scientists should also discuss the possibility of new systems to promote timely debate, for example dedicated journals, quick publication, open access and parallel commentaries.

To speed up publications dealing with clinical trials in rare disease and also to facilitate debate around such results requires a step-change in how trial data is made accessible. In effect this would require journals to adopt, for example, a different category of publication and to review and publish clinical trial data on a discrete track from other academic submissions.

## Broader issues on communication around clinical trials

A related point raised at the PEC by parents and patients concerns the perceived lack of openness of pharma with investigators and participants. In many cases trial results are not communicated or at best only aggregate results are released to clinicians and to patient organisations who have helped to fund the research or to set up a particular clinical trial (www.clinicaltrials.gov). This widespread concern is often expressed in terms of the right of those who have a direct interest in gaining access to timely, clear and appropriately detailed information about trial results including individual results where this is appropriate. This point is not new but has probably not been supported by the underpinning ethical arguments that have been elaborated in support of the related rights of research participants. There is wide international agreement that participation in clinical trials should be founded on adequately informed consent. The PEC’s perspective on this issue is that the principle of informed participation should be extended to a right to information after participation, since this is a reasonable extension of the principle of respect for autonomy which is implicit within informed consent 26 27 21 28 .

Some of those who raised this concern with the PEC asked whether the TREAT-NMD network could bring some leverage to bear on pharma who were seeking to access TREAT-NMD’s Global Patient Registry with an intention of running a clinical trial. While such stringent measures have strong moral support the PEC reflected that there was a risk that applying pressure on pharma might be counter productive and that much more could be gained by fostering a collegiate and collaborative approach between stakeholders, with clear voluntary agreements to conform to the required ethical standards, as other organisations have done (see Eurordis, www.eurordis.org), without first resort to mandatory formal agreements.

Compared to common diseases, there exists general agreement that RDs require dedicated regulatory issues for orphan drug designation and trial design (www.eurordis.org). Within the PEC it emerged that the role of patient associations and advocacy groups is fundamental as a bridge of communication between patients and pharma. Patient associations may be involved in trial design and even be signatories of trial contract agreements. Their role should serve to improve dialogue and communication between pharma, patients and their families, contributing to a better understanding of research and a higher quality of informed consent 29 .

## Overcoming barriers to the organisation of clinical trials: registries

Though SMA and DMD are among the more frequently occurring RDs, patients are dispersed across the world, which makes it difficult to organise a clinical trial. RDs are much less likely to benefit from research and healthcare development processes, which are designed with common diseases in mind. Yet in the interests of justice people with rare disease should be given the same opportunity to benefit from health research and treatments as those people with common disorders.

Prior to TREAT-NMD, patient organisations often in collaboration with academic centres, had made significant progress in developing and maintaining disease registries with a view to providing feasibility data for clinical trials. However, the effort was often fragmented and in order to facilitate accelerated research and development for RD data needs to be concentrated and standardised. Patient registries are crucial to achieving this. Patient recruitment through registries for RD clinical trials also raises particular ethical issues requiring good governance, oversight and audit of the registry 30 .

TREAT-NMD’s contribution has been to draw these separate initiatives together into a global disease registry with a validated and standardised approach to data collection, including a record of gene mutations which has become vital data in the current era of personalised, mutation specific therapies 30 31 . The PEC has proved an invaluable resource to the Global Registry Oversight Committee, the body responsible for the governance and strategic management of the global registry. In addition to some PEC members having dual membership on the Oversight Committee the whole of the PEC has been available as a sounding-board for ethical issues arising out of the establishment and running of the global registry. Both the PEC and the Oversight Committee drew upon the excellent resources created by Eurordis (http://www.eurordis.org/) giving practical guidance on the creation and maintenance of patient registries as well as advice on the ethical and legal governance of patient data 32 33 .

However it is clear that, despite the growing body of expertise in this area, there is a need to reflect upon and where necessary revise governance arrangements and remain responsive to the concerns of parents and patients when, in an act of trust they place their data and their hopes in the hands of others. A reflection of some of the anxieties patients and their families have in these contexts is reflected in one question that seems to be a recurrent theme in this context, namely that of ownership, of the registry, of the data, and by implication control over its use. Although the issue is comprehensively discussed in the literature and published guidelines (http://www.ukbiobank.ac.uk/docs/EGF20082_000.pdf) 34 35 , it is clear that part of the duty of care owed to registry participants is to offer open reassurance, to be responsive to queries, and to ensure, particularly in the initial informed consent process, that such matters are dealt with sensitively and honestly. As a guarantee that the privacy related rules are met, all registries should have a specific ethics committee, which monitors the flow of data into and out of the registry.

On the theme of good communication the PEC also supported the Global Registry in its efforts to find a clear and fair way of communicating with registry participants. The PEC strongly advised the separation of the role of the registry from that of trial co-ordinators to avoid the registry being seen as a recruiting agency. It was suggested that all registry participants should receive updates on the activities of the registry, but that more detailed information about specific clinical trials should only be issued to registry participants who met the inclusion criteria for the particular study; with a further option for patients to be able to opt out of "information only" letters (templates for these letters are available at www-treat-nmd.eu). Since the Global registry only contains anonymised data, the responsibility to determine the final form of communication with participants falls to participating national registries. At every point however the PEC was concerned with supporting the autonomy and rights of patients to have accurate, detailed and current information.

## Relationships with pharmaceutical industry

The establishment of the Global registry is one of the contexts in which discussion of the ethical issues related to collaboration with pharmaceutical companies has arisen. The PEC reflected on the view endorsed widely across TREAT-NMD that mutually beneficial relationships with industry should not be jeopardised by adopting too restrictive an approach to the genuine interests of industry to develop novel therapies. However this was balanced by the necessity to respect the wider rights and interests of patients in the ethical management of their data. It was recognised that here is a need to be open and honest with industry, to adopt procedural transparency without compromising commercially sensitive information but with an expectation that there is an open information flow between industry, clinicians and patients. The fact that a mutual co-operation between patients and industry is necessary should not result in an ethical approach dictated by a form of "prisoner’s dilemma", where both parties need the co-operation of the other in order to maximise self interest, but do so in an atmosphere of mistrust and lack of communication. A more desirable model morally, is one based upon mutual respect and understanding for which effective communication is a prerequisite. The broad principles offered by the PEC to guide relations with industry are summarised in Table 2, the PEC membership and desired qualities/ experiences are listed in Table 3.

**Table 2. The Principles offered by the PEC to guide relations with industry:** 

The broad ethical principles identified by the PEC in the context of patient registries and relationships with industry
1. Consent – any collation and use of patient data should be premised upon the provision of high quality information either directly to the patient/family or to the registry ‘owner’ e.g. Patient organisation.
2. All use of data should conform to the principles of informed consent
3. In order to maximise best use of patient data e.g. to support the development of further research, relations with industry ought to be conducted on the basis of open access: making data available to all parties who satisfy the oversight committee requirements and , transparency about who is interested in the data and for what purposes (in dialogue with industry partners as to what is reasonable in terms of commercial confidentiality)
4. Independent advice/scrutiny should be available to patients / patient organisations on the implications of granting access for industry to patient data.
5. Confidentiality- respecting the interests of industry on matters of commercial sensitivity where such measure can be reasonably justified and do not conflict with point 6.
6. Any arrangements/ partnerships with industry should be least restrictive of individual rights of patient participants.

**Table 3. PEC membership and desired qualities/ experience:** 

Since the PEC was not an executive committee it was open to members who were qualified by experience and were able to declare no conflict of interest.	
Chair	• Philosopher and bioethicist with experience of research ethics at national and international level.
Vice Chair	• Parent, senior member of parent/patient organisation.
Professional members	• Senior Clinicians with a clinical/research interest in NMDs, Genetics/Bio-scientists actively researching NMDs and potential therapy. • Academic lawyer with an interest in the ethical and legal aspects of children, Bioethicist. • Social scientist. • Parent with professional experience of regulatory affairs and industry.
Lay members	• Parents involved in patient/parent organisations.
Membership was drawn from Northern, Southern and Eastern Europe.	

## Personalised medicine and mutation specific trials: ensuring genetic molecular diagnosis

An important piece of data required by the Global registry pro-forma is the recording of the patient’s genetic diagnosis. The need for disease specific mutations is with an eye to the potential benefit from personalised medicine such as exon skipping or premature stop codon drugs for DMD, in which an accurate genetic diagnosis is essential 30 . Accurate detection in large genes with high allele heterogeneity is complex and requires specific expertise and equipment. This raises questions regarding the economic aspects of molecular testing as well as further ethical concerns if the aspiration of global treatment is to be realised.

The premise that all people with neuromuscular disorders (and other RDs) have a right to treatment and thus a right that appropriate clinical trials happen was the starting point for the PEC’s opinion. However, diagnostic facilities are not equally available in Europe which makes the development of diagnostic capacity, which will in turn support the development of clinical trials, a priority.

Two projects related to TREAT-NMD should help to facilitate this. The FP7- funded NMD-Chip project (http://www.nmd-chip.eu/) has developed new high throughput tools to improve the speed and accuracy of the diagnostic tests while reducing costs. From this project two major questions were brought to the attention of the PEC, namely integrity issues regarding use of DNA reference material for validation of the new tool for molecular diagnosis of NMDs, and how to deal with incidental findings of mutations not related to the original reason to perform the DNA diagnosis. The BIO-NMD project (http://www.bio-nmd.eu/) is aiming to discover novel, disease-specific, biomarkers with a view to improving diagnostics at lower costs. In addition TREAT-NMD and EUROGENTEST have jointly published guidelines for the genetic diagnosis of DMD, the most common muscular dystrophy affecting children 36. The ethics and governance structures of these projects have both benefitted from access to the TREAT-NMD PEC.

Due to the rarity of NMDs, larger clinical trials often involve multiple centres in multiple countries. However, these multicentre trials are hampered by the fact that care standards differ for different countries. The TREAT-NMD related CARE-NMD project aims to address this, with a special focus on optimising care in Eastern European countries (http://en.care-nmd.eu/).

The potential availability of therapies for DMD in the near future raised discussions in the PEC about the need to reassess neonatal screening. The Wilson and Jungner criteria for screening state that screening should be limited to diseases for which a treatment is available 37 , which is not (yet) the case for DMD. However, recently the US neonatal screening results have been published providing answers on that but also raising ethical questions 38 39 . Based on that, PEC members have argued further that the Wilson and Jungner approach rests upon the principle of the child’s best interests. Thus a case could be made to introduce screening for DMD now for reasons that include a recognition of the impact of the ‘diagnostic odyssey’ where diagnosis is not confirmed for, on average, 2 years after the first concerns are raised. Parents have claimed that early diagnosis would have allowed them to be better parents thus furthering the interests of the child. A second and perhaps more direct consideration takes into account the potential benefit from early use of steroids in prolonging ambulation and improving respiratory function 40 .

## The child’s view: involving children in decision making around trial participation

The ethics of clinical research are based on several known guidelines in which the principles of autonomy, beneficence, non-maleficence, and fairness remain constant 35 41 .

How such principles are honoured can be open to interpretation and when children are the subjects of clinical trials these principles become more complicated, indeed some legal guidance precludes the child’s consent to a clinical trial presuming the children’s lack of autonomy and inherent vulnerability.

For children under 16 years of age parental/guardian consent is a requirement. Several strands of discussion at the PEC dealt with related issues of consent and children’s participation in research.

Decisions about trial participation are taken by the parents or adults legally responsible for the child and guidelines for the conduct of research in clinical trials generally do not recognise children’s autonomy. Some guidelines explicitly recommend that assent to participation in trials should be sought from children (CIOMS^42^ and Helsinki^43^ ) and others that the child should not be involved if they object or appear to object 44 . However, there is no legal obligation for investigators to actually seek a child’s assent and few guidelines on how one might go about doing so 45 46 47 . In addition, it is unclear how widespread the practice of seeking assent is.

## Conclusions

New therapies in rare NMDs raised specific ethical issues that are not fully covered by commonly used ethical guidelines. An appropriate ethical clarification of these issues needs an institutional capacity to identify, see and evaluate them in their concrete contexts. To do this needs the experiences of families who are acquainted with the disease. Therefore, ethical expertise needs to be organised not only inter-disciplinarily (as is common in research ethics committees) but also include experiential knowledge through the inclusion of patient representatives.

The TREAT-NMD PEC addressed some of the main issues related to rare NMDs care, diagnosis, treatments and ethics, possibly representing a suitable model for addressing similar issues in the context of other Rare Diseases.

## Competing Interests

The authors have declared that no competing interests exist. Annemieke Aartsma-Rus is employed by LUMC, which has patent applications on exon skipping and as co-inventor on some of these patents AAR is entitled to a share of royalties. Alessandra Ferlini is PI of Prosensa and GSK trials on exon skipping in Duchenne Muscular Dystrophy. Prof Francesco Muntoni has served on scientific advisory boards for Acceleron Pharma, Genzyme, AVI BioPharma, Debiopharma Group, GSK; Prosensa, Servier and Santhera Pharmaceutical and he is receiving funding for trials from GSK and the British Heart Foundation and has received funding for trials from AVI BioPharma, Trophos and PTC. All Authors have received research grants from many Funding Agencies including EU and serve on editorial Boards of several scientific journals.

## Correspondence

Pauline McCormack. Email: pauline.mccormack@newcastle.ac.uk

Alessandra Ferlini. Email: fla@unife.it

## References

[1] Council of Europe (1999). Regulation on orphan medicinal products. Brussels, EC. (www.coe.int)

[2] European Commission (2010). European action in the field of Rare Diseases. H. a. C. D. General. Luxembourg, EC.(http://ec.europa.eu/health/rare_diseases/docs/eucerd2011_report_state_of_art_raredis_activities_2.pdf)

[3] van Engelen BG, van Veenendaal H, van Doorn PA, Faber CG, van der Hoeven JH, Janssen NG, Notermans NC, van Schaik IN, Visser LH, Verschuuren JJ. The Dutch neuromuscular database CRAMP (Computer Registry of All Myopathies and Polyneuropathies): development and preliminary data. Neuromuscul Disord. 2007 Jan;17(1):33-7. PubMed PMID:17141501. 17141501 10.1016/j.nmd.2006.09.017

[4] Norwood FL, Harling C, Chinnery PF, Eagle M, Bushby K, Straub V. Prevalence of genetic muscle disease in Northern England: in-depth analysis of a muscle clinic population. Brain. 2009 Nov;132(Pt 11):3175-86. PubMed PMID:19767415. 19767415 10.1093/brain/awp236 PMC4038491

[5] van Deutekom JC, Janson AA, Ginjaar IB, Frankhuizen WS, Aartsma-Rus A, Bremmer-Bout M, den Dunnen JT, Koop K, van der Kooi AJ, Goemans NM, de Kimpe SJ, Ekhart PF, Venneker EH, Platenburg GJ, Verschuuren JJ, van Ommen GJ. Local dystrophin restoration with antisense oligonucleotide PRO051. N Engl J Med. 2007 Dec 27;357(26):2677-86. PubMed PMID:18160687. 18160687 10.1056/NEJMoa073108

[6] Kinali M, Arechavala-Gomeza V, Feng L, Cirak S, Hunt D, Adkin C, Guglieri M, Ashton E, Abbs S, Nihoyannopoulos P, Garralda ME, Rutherford M, McCulley C, Popplewell L, Graham IR, Dickson G, Wood MJ, Wells DJ, Wilton SD, Kole R, Straub V, Bushby K, Sewry C, Morgan JE, Muntoni F. Local restoration of dystrophin expression with the morpholino oligomer AVI-4658 in Duchenne muscular dystrophy: a single-blind, placebo-controlled, dose-escalation, proof-of-concept study. Lancet Neurol. 2009 Oct;8(10):918-28. PubMed PMID:19713152. 19713152 10.1016/S1474-4422(09)70211-X PMC2755039

[7] Chakrapani A, Vellodi A, Robinson P, Jones S, Wraith JE. Treatment of infantile Pompe disease with alglucosidase alpha: the UK experience. J Inherit Metab Dis. 2010 Dec;33(6):747-50. PubMed PMID:20865334. 20865334 10.1007/s10545-010-9206-3

[8] London AJ, Kimmelman J, Emborg ME. Research ethics. Beyond access vs. protection in trials of innovative therapies. Science. 2010 May 14;328(5980):829-30. PubMed PMID:20466907. 20466907 10.1126/science.1189369 PMC4516403

[9] Mendell JR, Rodino-Klapac LR, Rosales XQ, Coley BD, Galloway G, Lewis S, Malik V, Shilling C, Byrne BJ, Conlon T, Campbell KJ, Bremer WG, Taylor LE, Flanigan KM, Gastier-Foster JM, Astbury C, Kota J, Sahenk Z, Walker CM, Clark KR. Sustained alpha-sarcoglycan gene expression after gene transfer in limb-girdle muscular dystrophy, type 2D. Ann Neurol. 2010 Nov;68(5):629-38. PubMed PMID:21031578. 21031578 10.1002/ana.22251 PMC2970162

[10] Goemans NM, Tulinius M, van den Akker JT, Burm BE, Ekhart PF, Heuvelmans N, Holling T, Janson AA, Platenburg GJ, Sipkens JA, Sitsen JM, Aartsma-Rus A, van Ommen GJ, Buyse G, Darin N, Verschuuren JJ, Campion GV, de Kimpe SJ, van Deutekom JC. Systemic administration of PRO051 in Duchenne's muscular dystrophy. N Engl J Med. 2011 Apr 21;364(16):1513-22. PubMed PMID:21428760. 21428760 10.1056/NEJMoa1011367

[11] Cirak S, Arechavala-Gomeza V, Guglieri M, Feng L, Torelli S, Anthony K, Abbs S, Garralda ME, Bourke J, Wells DJ, Dickson G, Wood MJ, Wilton SD, Straub V, Kole R, Shrewsbury SB, Sewry C, Morgan JE, Bushby K, Muntoni F. Exon skipping and dystrophin restoration in patients with Duchenne muscular dystrophy after systemic phosphorodiamidate morpholino oligomer treatment: an open-label, phase 2, dose-escalation study. Lancet. 2011 Aug 13;378(9791):595-605. PubMed PMID:21784508. 21784508 10.1016/S0140-6736(11)60756-3 PMC3156980

[12] Pareyson D, Reilly MM, Schenone A, Fabrizi GM, Cavallaro T, Santoro L, Vita G, Quattrone A, Padua L, Gemignani F, Visioli F, Laurà M, Radice D, Calabrese D, Hughes RA, Solari A. Ascorbic acid in Charcot-Marie-Tooth disease type 1A (CMT-TRIAAL and CMT-TRAUK): a double-blind randomised trial. Lancet Neurol. 2011 Apr;10(4):320-8. PubMed PMID:21393063. 21393063 10.1016/S1474-4422(11)70025-4 PMC3154498

[13] Nigro V, Aurino S, Piluso G. Limb girdle muscular dystrophies: update on genetic diagnosis and therapeutic approaches. Curr Opin Neurol. 2011 Oct;24(5):429-36. PubMed PMID:21825984. 21825984 10.1097/WCO.0b013e32834aa38d

[14] Bowles DE, McPhee SW, Li C, Gray SJ, Samulski JJ, Camp AS, Li J, Wang B, Monahan PE, Rabinowitz JE, Grieger JC, Govindasamy L, Agbandje-McKenna M, Xiao X, Samulski RJ. Phase 1 gene therapy for Duchenne muscular dystrophy using a translational optimized AAV vector. Mol Ther. 2012 Feb;20(2):443-55. PubMed PMID:22068425. 22068425 10.1038/mt.2011.237 PMC3277234

[15] Woods S, McCormack P. DISPUTING THE ETHICS OF RESEARCH: THE CHALLENGE FROM BIOETHICS AND PATIENT ACTIVISM TO THE INTERPRETATION OF THE DECLARATION OF HELSINKI IN CLINICAL TRIALS. Bioethics. 2012 Feb 2. PubMed PMID:22296646. 22296646 10.1111/j.1467-8519.2011.01945.x

[16] Naldini L. Ex vivo gene transfer and correction for cell-based therapies. Nat Rev Genet. 2011 May;12(5):301-15. PubMed PMID:21445084. 21445084 10.1038/nrg2985

[17] Prayle AP, Hurley MN, Smyth AR. Compliance with mandatory reporting of clinical trial results on ClinicalTrials.gov: cross sectional study. BMJ. 2012 Jan 3;344:d7373. PubMed PMID:22214756. 22214756 10.1136/bmj.d7373

[18] Ross JS, Tse T, Zarin DA, Xu H, Zhou L, Krumholz HM. Publication of NIH funded trials registered in ClinicalTrials.gov: cross sectional analysis. BMJ. 2012 Jan 3;344:d7292. PubMed PMID:22214755. 22214755 10.1136/bmj.d7292 PMC3623605

[19] Hurley MN, Prayle AP, Smyth AR. Delayed publication of clinical trials in cystic fibrosis. J Cyst Fibros. 2012 Jan;11(1):14-7. PubMed PMID:21889426. 21889426 10.1016/j.jcf.2011.08.004 PMC3267039

[20] Schott G, Pachl H, Limbach U, Gundert-Remy U, Lieb K, Ludwig WD. The financing of drug trials by pharmaceutical companies and its consequences: part 2: a qualitative, systematic review of the literature on possible influences on authorship, access to trial data, and trial registration and publication. Dtsch Arztebl Int. 2010 Apr;107(17):295-301. PubMed PMID:20490338. 20490338 10.3238/arztebl.2010.0295 PMC2872821

[21] Henschel AD, Rothenberger LG, Boos J. Randomized clinical trials in children--ethical and methodological issues. Curr Pharm Des. 2010;16(22):2407-15. PubMed PMID:20513232. 20513232 10.2174/138161210791959854

[22] Koelch M, Fegert JM. Ethics in child and adolescent psychiatric care: An international perspective. Int Rev Psychiatry. 2010;22(3):258-66. PubMed PMID:20528655. 20528655 10.3109/09540261.2010.485979

[23] Bushby K, Connor E. Clinical outcome measures for trials in Duchenne muscular dystrophy: report from International Working Group meetings. Clin Investig (Lond). 2011 Sep;1(9):1217-1235. 10.4155/cli.11.113 PMC3357954 22639722

[24] Klein A, Jungbluth H, Clement E, Lillis S, Abbs S, Munot P, Pane M, Wraige E, Schara U, Straub V, Mercuri E, Muntoni F. Muscle magnetic resonance imaging in congenital myopathies due to ryanodine receptor type 1 gene mutations. Arch Neurol. 2011 Sep;68(9):1171-9. PubMed PMID:21911697. 21911697 10.1001/archneurol.2011.188

[25] Lundh A, Krogsbøll LT, Gøtzsche PC. Access to data in industry-sponsored trials. Lancet. 2011 Dec 10;378(9808):1995-6. PubMed PMID:22153200. 22153200 10.1016/S0140-6736(11)61871-0

[26] Coultas D. Ethical considerations in the interpretation and communication of clinical trial results. Proc Am Thorac Soc. 2007 May;4(2):194-8; discussion 198-9. PubMed PMID:17494731. 17494731 10.1513/pats.200701-007GC

[27] Regulation (EC) No 1901/2006 of the european parliament and of the council of 12 december 2006 on medical products for paediatric use amending regulation (ECC) No 1768/92, Directive 2001/20/EC, Directive 2001/83/EC and Regulation (EC) No 726/2004.

[28] Aartsma-Rus A. The risks of therapeutic misconception and individual patient (n=1) "trials" in rare diseases such as Duchenne dystrophy. Neuromuscul Disord. 2011 Jan;21(1):13-5. PubMed PMID:21051233. 21051233 10.1016/j.nmd.2010.09.012

[29] Woods S, Hagger LE, McCormack P. Therapeutic Misconception: Hope, Trust and Misconception in Paediatric Research. Health Care Anal. 2012 Feb 18. PubMed PMID:22350619. 22350619 10.1007/s10728-012-0201-8

[30] Muntoni F, Wells D. Genetic treatments in muscular dystrophies. Curr Opin Neurol. 2007 Oct;20(5):590-4. PubMed PMID:17885450. 17885450 10.1097/WCO.0b013e3282efc157

[31] Arechavala-Gomeza V, Anthony K, Morgan J, Muntoni F. Antisense oligonucleotide-mediated exon skipping for Duchenne muscular dystrophy: progress and challenges. Curr Gene Ther. 2012 Jun;12(3):152-60. PubMed PMID:22533380. 22533380 10.2174/156652312800840621

[32] Moliner AM. Creating a European Union framework for actions in the field of rare diseases. Adv Exp Med Biol. 2010;686:457-73. PubMed PMID:20824460. 20824460 10.1007/978-90-481-9485-8_25

[33] Rubinstein YR, Groft SC, Chandros SH, Kaneshiro J, Karp B, Lockhart NC, Marshall PA, Moxley RT 3rd, Pollen GB, Miller VR, Schwartz J. Informed consent process for patient participation in rare disease registries linked to biorepositories. Contemp Clin Trials. 2012 Jan;33(1):5-11. PubMed PMID:22036955. 22036955 10.1016/j.cct.2011.10.004 PMC4464841

[34] Horton R. Trial registers: protecting patients, advancing trust. Lancet. 2006 May 20;367(9523):1633-5. PubMed PMID:16714167. 16714167 10.1016/S0140-6736(06)68709-6

[35] Davidson AJ, O'Brien M. Ethics and medical research in children. Paediatr Anaesth. 2009 Oct;19(10):994-1004. PubMed PMID:19709376. 19709376 10.1111/j.1460-9592.2009.03117.x

[36] Abbs S, Tuffery-Giraud S, Bakker E, Ferlini A, Sejersen T, Mueller CR. Best practice guidelines on molecular diagnostics in Duchenne/Becker muscular dystrophies. Neuromuscul Disord. 2010 Jun;20(6):422-7. PubMed PMID:20466545. 20466545 10.1016/j.nmd.2010.04.005

[37] Ross LF. Screening for conditions that do not meet the Wilson and Jungner criteria: the case of Duchenne muscular dystrophy. Am J Med Genet A. 2006 Apr 15;140(8):914-22. PubMed PMID:16528755. 16528755 10.1002/ajmg.a.31165

[38] Mendell JR, Shilling C, Leslie ND, Flanigan KM, al-Dahhak R, Gastier-Foster J, Kneile K, Dunn DM, Duval B, Aoyagi A, Hamil C, Mahmoud M, Roush K, Bird L, Rankin C, Lilly H, Street N, Chandrasekar R, Weiss RB. Evidence-based path to newborn screening for Duchenne muscular dystrophy. Ann Neurol. 2012 Mar;71(3):304-13. PubMed PMID:22451200. 22451200 10.1002/ana.23528

[39] Ferlini A. Neuromuscular disease: Muscular dystrophy--something new on God's green earth? Nat Rev Neurol. 2012 Mar 27;8(5):247-9. PubMed PMID:22450509. 22450509 10.1038/nrneurol.2012.41

[40] Merlini L, Gennari M, Malaspina E, Cecconi I, Armaroli A, Gnudi S, Talim B, Ferlini A, Cicognani A, Franzoni E. Early corticosteroid treatment in 4 Duchenne muscular dystrophy patients: 14-year follow-up. Muscle Nerve. 2012 Jun;45(6):796-802. PubMed PMID:22581531. 22581531 10.1002/mus.23272

[41] Beauchamp and Childress. 2009. Principles of Biomedical Ethics. Sixth edition. Oxford University Press, New York NY

[42] Council for International Organizations of Medical Sciences (CIOMS). 2002. International Ethical Guidelines for Biomedical Research Involving Human Subjects. 3rd edn. Geneva: CIOMS: 63. 14983848

[43] World Medical Association. 1964. World Medical Association Declaration of Helsinki. Ethical Principles for Medical Research Involving Human Subjects. Ferney-Voltaire, France: World Medical Association.

[44] UK Medical Research Council, Medical Research Involving Children, 2004 http://www.mrc.ac.uk/Utilities/Documentrecord/index.htm?d=MRC002430

[45] Hagger, L. (2009) The Child as Vulnerable Patient: Protection and Empowerment, Abingdon: Ashgate

[46] Cave E. Seen but not heard? Children in clinical trials. Med Law Rev. 2010 Winter;18(1):1-27. PubMed PMID:20129995. 20129995 10.1093/medlaw/fwp024

[47] Alderson P, Morrow V (2011) The ethics of research with children and young people: a practical handbook. London: Sage Publications.

